# Supplementary material for: Correlation of lipoprotein-associated phospholipase A2 and cerebral microbleeds in patients with acute ischaemic stroke
Source: BMC Neurol. 2022 Dec 14;22:482. doi: 10.1186/s12883-022-03000-w (PMC9749267; doi:10.1186/s12883-022-03000-w)
Supplement: Supplementary file 1 — Additional file 1. [file 12883_2022_3000_MOESM1_ESM.docx]

**Correlation of Lipoprotein-Associated Phospholipase A2 and Cerebral Microbleeds in Patients with Acute Ischaemic Stroke**

Xiaojiu Zhang^1,2^·Lu Liu^1^·Nan Jiang^3^·Yufeng Liu^1^·Qing Wang^1^·Xiaohong Tang^2^·Qijin Zhai^1^·Liandong Zhao^1^

1. Department of Neurology, The Afliated Huai’an Hospital of Xuzhou Medical University, No.62, South Huai’an Road, Huai’an 223002, Jiangsu, China
2. Department of Neurology,Hongze People's Hospital, Hongze 223100, Jiangsu, China
3. Department of Neurology, Lianshui people’s Hospital Affiliated to Kangda College of Nanjing Medical University,Lianshui 223400, Jiangsu, China

Xiaojiu Zhang, Lu Liu, and Nan Jiang have contributed equally to this work

Xiaojiu Zhang zxj7418695@ 163.com the first author

[Lu Liu ll18796201519@163.com](mailto:刘露ll18796201519@163.com) Co-first author

[Jiang Nan 13912068234@139.com](mailto:姜楠13912068234@139.com) Co-first author

Yufeng Liu lyf996226@ 163.com the second author

[Qing Wang wangqing@163.com](mailto:王清wangqing@163.com) the third author

Xiaohong Tang txh 13915144630@ 163.com the fourth author

Qijin Zhai [qjzhai1983@163.com](mailto:qjzhai1983@163.com) Co-corresponding author

Liandong Zhao zld20220429@ 163.com Corresponding author

## Abstract

**Background and aims** Cerebral microbleeds (CMBs) increase the risk of stroke occurrence and recurrence,and affect the prognosis of stroke patients. Therefore, identifying biological markers that predict CMBs after stroke is urgently needed. This study explored whether high levels of lipoprotein-associated phospholipase A2(Lp-PLA2) are associated with an increased risk of CMBs in patients with acute ischaemic stroke (AIS).

**Methods** From April 2020 to October 2021, we enrolled 242 patients with AIS. At admission, the plasma levels of Lp-PLA2 were measured in all patients as well as the number of CMBs and white matter lesions.According to the results of the Susceptibility Weighted Imaging (SWI), the patients were divided into a CMB group and a no-CMB group. The groups were compared with univariate and multivariate analyses to clarify the correlation between Lp-PLA2 levels and CMBs, and the optimal cut-off value of Lp-PLA2 that predicted CMBs was determined from the receiver-operating characteristic curve.

**Results** CMBs were detected in 71 (29.3%) of the 242 AIS patients. The median Lp-PLA2 level was 182.79 ng/ml. Using the 1^st^ quartile of Lp-PLA2 levels (the lowest levels) as the reference group, univariate logistic regression analysis showed that individuals in the 4^th^ quartile (the highest levels) had a higher risk of CMBs (odds ratio [OR] = 1.460, 95% confidence interval [CI]: 1.188-1.795, *P* = 0.000). This correlation persisted after adjusting for relevant risk factors (OR = 1.370, 95% CI: 1.096-1.713, *P* = 0.006). The optimal cut-off value of Lp-PLA2 that predicted the occurrence of CMBs was 184.36 ng/ml; at this threshold, the sensitivity was 69.0%, and the specificity was 60.2%.

**Conclusions** Our data suggest that a high level of Lp-PLA2 in patients with AIS is a potential risk factor for CMBs.

**Keywords** Lipoprotein-associated phospholipase A2 · Cerebral microbleeds · Acute ischaemic stroke · Risk factor

## Introduction

Cerebral microbleeds (CMBs) are subclinical damage of brain parenchyma characterized by microvascular lesions and microbleeds in the brain[1]. With advances in imaging technology, especially the development of susceptibility weighted imaging (SWI), the ability to detect CMBs has increased. Studies have shown that the proportion of CMBs in patients with ischemic stroke is more than 5 times that of healthy people[2].

Generally, CMBs have no obvious clinical symptoms or signs, but studies have confirmed thatthat CMBs not only predict the risks of ischaemic stroke occurrence and recurrence[3, 4] but also increase the risk of intracerebral haemorrhage in patients receiving antiplatelet therapy[5] and the risk of symptomatic intracerebral haemorrhage after thrombolytic therapy[6]. As the presence of CMBs seriously affects the occurrence, recurrence and prognosis of ischaemic stroke, it is highly important to identify the risk factors for CMBs.

In recent years, many studies have shown that CMBs are closely related to inflammatory factors such as high-sensitivity C-reactive protein (hs-CRP), interleukin (IL)-6, IL-18 and tumour necrosis factor alpha (TNF-α)[7, 8]. Therefore, there may be a correlation between CMBs and inflammatory markers. Unlike systemic inflammatory factors such as hs-CRP and ILs, lipoprotein associated phospholipase A2 (Lp-PLA2) is a vascular-specific inflammatory factor with a relatively stable concentration that is not affected by infection. The level of Lp-PLA2 thus directly and accurately reflects the extent of intravascular inflammation[9]. However, few studies have explored the correlation between Lp-PLA2 levels and CMBs.

Therefore, the aim of this study was to investigate the correlation between Lp-PLA2 levels and CMBs in patients with acute ischaemic stroke (AIS) and to determine the optimal cut-off value of Lp-PLA2 that predicts CMBs.

## Materials and Methods

### Study Population

The clinical data of 242 AIS patients admitted to our hospital from April 2020 to October 2021 were collected and analysed. The inclusion criteria for patients were as follows: (1) met the diagnostic criteria of the World Health Organization (WHO) for AIS confirmed by craniocerebral magnetic resonance imaging (MRI) or computed tomography (CT) scans[10]; (2) had an AIS onset time within 6–48 hours; (3) age ≥18 years; and (4) had complete clinical data available. The exclusion criteria were as follows: (1) emergency intravenous thrombolysis or endovascular intervention; (2) previous history of intracerebral haemorrhage, subarachnoid haemorrhage, brain trauma, or brain tumour; (3) severe infection, autoimmune disease, malignant tumour or blood system disease; and (4) severe dysfunction of the heart, liver, or kidney.

All of the participants signed an informed form to participate in the study. The study design was approved by the Medical Ethics Committee of Huai'an Hospital affiliated to Xuzhou Medical University.

### Collection of Baseline Data

Data on demographic and clinical characteristics were collected at admission, including age, sex, smoking status, alcohol consumption, history of related diseases(diabetes, hypertension, stroke, atrial fibrillation and coronary heart disease), premorbid medications (antithrombotics, anticoagulants and statins), baseline systolic and diastolic blood pressure, National Institutes of Health Stroke Scale (NIHSS) score, and Alberta Stroke Program Early CT (ASPECT) score.  Laboratory data, including hs-CRP, lipid profle results, fasting plasma glucose(FPG), homocysteine(HCY), blood coagulation indicators and creatinine (Cr), were also recorded. All tests were performed with a fully automated biochemical analyser (Beckman 5811, Germany).

### Lp-PLA2 Concentration Assessment

Fasting venous blood was collected from all patients within 24 h of admission and placed in EDTA-treated tubes. Blood samples were centrifuged at 1,500 r/min for 10 minutes, and the separated plasma was stored at 4°C. Next, the plasma levels of Lp-PLA2 were measured by magnetic microparticle chemiluminescence in an MQ60 series automatic immune analyser according to the kit’s instructions. Reagents were provided by Beijing Rejing Biotechnology Co., Ltd.

### MRI Scans

Cranial MRI was performed using a 3.0T MRI scanner (General Electric Medical Systems, USA). All patients completed the following sequences within 72 hours of admission: T1-weighted imaging *(*repetition time [TR], 1,750 ms; echotime [TE], 24 ms; fifield of view [FOV], 24×18 cm; matrix, 320×224; slice thickness, 5 mm), T2- weighted imaging (TR, 4,841 ms; TE, 102 ms; FOV, 24×24 cm; matrix, 256×256; slice thickness, 5 mm), fluid-attenuated inversion recovery (FLAIR) (TR, 9,000 ms; TE, 130 ms; FOV, 24×24 cm; matrix, 256×192; slice thickness, 5 mm), diffusion weighted imaging (DWI) (TR, 4,880 ms; TE, 65 ms; b-value, 0/1000;FOV, 24×24 cm; matrix, 160×130; slice thickness, 5 mm), SWI (TR, 85 ms; TE, 45 ms;flip angle,15°;FOV, 24×22 cm; matrix, 384×320; slice thickness, 2 mm;slice gap, 0 mm). All imaging results were observed and assessed by two experienced neuroradiologists. If disagreements arose, the two doctors discussed the case until they reached an agreement.

The positive diagnostic criteria for CMBs in SWI results were as follows[11]: round or oval hypointense signals with a diameter of 2~5 mm (maximum of no more than 10 mm), with clear borders, without oedema around the focus, and excluding calcium or iron deposition, vascular flow void effect, diffuse axonal injury, cavernous haemangioma or other conditions with similar imaging manifestations.

White matter lesions (WMLs) were defined as high signal intensity in T2-weighted images and FLAIR sequences of cranial MRI results, equal or slight signal on T1-weighted images, and focuses that were punctate or patchy with blurred borders and diameters ≥ 5 mm[12].

WMLs were assessed according to the Fazekas score [13], which included two categories: periventricular hyperintensity (PVH) and deep white matter hyperintensity (DWMH) scores. (1) PVH scores were as follows: 0 for no lesions, 1 for cap or pencil-like thin-layer lesions, 2 for smooth halo lesions, and 3 for lesions extending to deep white matter; (2) DWMH scores were as follows: 0 for no lesions, 1 for spotted lesions, 2 for plaque-like partial fusion lesions and 3 for diffuse large fusion lesions. The final Fazekas score was calculated as the sum of the PVH and DWMH scores.

### Statistical Analysis

All statistical analyses were performed using SPSS version 25.0 (SPSS Inc., Chicago, IL, USA). Normally distributed data are expressed as the mean ± standard deviation (SD), and group comparisons were performed by two independent-sample *t* tests. Nonnormally distributed data are expressed as the median and interquartile range. Group comparisons were made with the Mann‒Whitney U test. Count data are expressed as the number and percentage (n, %), and group comparisons were performed using the chi-square test. Univariate and multivariate logistic regression analyses were used to evaluate the correlation between Lp-PLA2 levels and CMBs. A receiver-operating characteristic curve (ROC) was used to estimate the optimal cut-off value of Lp-PLA2 for predicting CMBs in patients with AIS, and the area under the curve (AUC), sensitivity and specificity of the ROC were evaluated. Statistical significance was established at *P* < 0.05 in all tests.

## Results

Of 242 patients with AIS were included, the age of them ranged from 42 to 90 years, with an average age of 67.52±9.50 years. Among these patients, 158 were male (65.3%) and 84 were female (34.7%), 87 (36%) had a history of smoking, and 76 (31.4%) had a history of alcohol consumption. In addition, 158 (65.3%) patients had hypertension, and 69 (28.5%) patients had diabetes mellitus. Furthermore, 48 (19.8%) patients took antiplatelet drugs, and 13 (5.4%) patients took anticoagulants.

The 242 subjects were divided into a CMB group and a no-CMB group according to the presence or absence of CMBs. As shown in Table 1, age, hypertension and diabetes, Fazekas score, total cholesterol (TC) and low-density lipoprotein cholesterol (LDL-C) levels, and HCY and Lp-PLA2 levels were significantly higher in the CMB group than in the no-CMB group (*P* < 0.05). Furthermore, there were no significant differences between the two groups in sex, history of smoking or drinking, history of coronary heart disease, atrial fibrillation or stroke, use of antithrombotics and anticoagulants, Hs-CRP, FPG or blood coagulation indicators (*P* > 0.05).

**Table 1** Comparison of general clinical data between the CMB and no-CMB groups

| Variables | CMB group  (n = 71) | No-CMB group  (n = 171) | *P* value |
| --- | --- | --- | --- |
| **Demographic data** |  |  |  |
| Age, years | 69.5±9.9 | 66.7±9.2 | 0.036 |
| Sex, male (%) | 48 (67.6) | 110 (64.3) | 0.626 |
| History of smoking (%) | 26 (36.6) | 61 (35.7) | 0.889 |
| History of drinking (%) | 24 (33.8) | 52 (30.4) | 0.605 |
| Hypertension (%) | 54 (76.1) | 104 (60.8) | 0.023 |
| Diabetes mellitus (%) | 27 (38.0) | 42 (24.6) | 0.035 |
| Coronary heart disease (%) | 16 (22.5) | 44 (25.7) | 0.600 |
| History of stroke (%) | 26 (36.6) | 58 (33.9) | 0.688 |
| Atrial fibrillation (%) | 9 (12.7) | 21 (12.3) | 0.932 |
| Use of anticoagulants (%) | 4 (5.6) | 9 (5.3) | 0.907 |
| Use of antithrombotics (%) | 16 (22.5) | 32 (18.7) | 0.497 |
| Use of statins (%) | 14 (19.7) | 25 (14.6) | 0.326 |
| **Clinical data** |  |  |  |
| Systolic blood pressure (mmHg) | 146.4±13.4 | 142.9±13.1 | 0.066 |
| Diastolic blood pressure (mmHg) | 87.9±7.9 | 86.2±8.4 | 0.144 |
| NIHSS score | 7 (4, 10) | 6 (3, 9) | 0.063 |
| ASPECT score | 7 (5, 8) | 7 (6, 9) | 0.175 |
| Fazekas score | 3 (2, 4) | 2 (1, 3) | 0.001 |
| **Laboratory data** |  |  |  |
| Hs-CRP (mg/L) | 2.83 (1.62, 4.42) | 2.46 (1.48, 4.14) | 0.525 |
| FPG (mmol/l) | 5.69 (5.23, 6.77) | 5.55 (5.0 6, 5.98) | 0.242 |
| TG (mmol/L) | 1.46 (0.78, 1.94) | 1.31 (0.70, 1.67) | 0.156 |
| TC (mmol/L) | 4.34±0.71 | 4.12±0.84 | 0.041 |
| LDL-C (mmol/L) | 2.64±0.45 | 2.51±0.46 | 0.047 |
| HDL (mmol/L) | 1.20±0.24 | 1.25±0.25 | 0.214 |
| PT (s) | 11.75±1.07 | 11.89±0.95 | 0.347 |
| APTT (s) | 31.23±3.32 | 30.55±3.38 | 0.152 |
| INR | 1.08±0.12 | 1.07±0.11 | 0.483 |
| FIB (g/L) | 2.79±0.80 | 2.75±0.83 | 0.707 |
| Cr (umol/l) | 76.43±11.84 | 77.87±17.98 | 0.465 |
| HCY (umol/l) | 16.31±3.68 | 14.34±4.14 | 0.001 |
| Lp-PLA2 (ng/ml) | 196.07±35.36 | 176.96±32.01 | 0.000 |

Abbreviations: CMBs, cerebral microbleeds; hs-CRP, high-sensitivity C-reactive protein; FPG, fasting plasma glucose; TG, triglycerides; TC, total cholesterol; LDL-C, low-density lipoprotein cholesterol; HDL, high-density lipoprotein; PT, prothrombin time; APTT, activated partial thromboplastin time; INR, International Normalized Ratio; FIB, fibrinogen; Cr, creatinine; HCY, homocysteine; Lp-PLA2, lipoprotein associated phospholipase A2.

The median Lp-PLA2 level at admission was 182.79 ng/ml. Lp-PLA2 levels were divided into quartiles: 1^st^ (< 158.56 ng/ml), 2^nd^ (158.56-182.79 ng/ml), 3^rd^  (182.79-204.67 ng/ml) and 4^th^ (> 204.67 ng/ml). Logistic regression analysis was used to evaluate the relationship between Lp-PLA2 levels and CMBs. As shown in Table 2, using the 1st quartile of Lp-PLA2 levels (the lowest levels) as the reference group, univariate logistic regression analysis showed that individuals in the 4th quartile (the highest levels) had a higher risk of CMBs (odds ratio [OR] = 1.460, 95% confidence interval [CI]: 1.188-1.795, *P* = 0.000). Moreover, this correlation persisted after adjusting for potential confounders such as age, hypertension, diabetes, Fazekas score, TC, LDL-C, and HCY (OR = 1.370, 95% CI: 1.096-1.713, *P* = 0.006). In addition, when Lp-PLA2 was used as a continuous variable for logistic regression analysis, the level of Lp-PLA2 was also correlated with CMBs after adjusting for the above risk factors(OR = 1.014, 95% CI: 1.005-1.024, *P* = 0.004).

**Table 2** Logistic regression analysis of the relationship between Lp-PLA2 levels and CMBs

| Lp-PLA2 level | OR | 95% CI | *P* value |
| --- | --- | --- | --- |
| Model 1 |  |  |  |
| 1^st^ quartile | Reference |  |  |
| 2^nd^ quartile | 1.010 | 0.636-1.603 | 0.966 |
| 3^rd^ quartile | 1.272 | 0.958-1.687 | 0.096 |
| 4^th^ quartile | 1.460 | 1.188-1.795 | 0.000 |
| Model 2 |  |  |  |
| 1^st^ quartile | Reference |  |  |
| 2^nd^ quartile | 0.923 | 0.559-1.523 | 0.753 |
| 3^rd^ quartile | 1.334 | 0.978-1.820 | 0.069 |
| 4^th^ quartile | 1.370 | 1.096-1.713 | 0.006 |
| Model 3 |  |  |  |
|  | 1.014 | 1.005-1.024 | 0.004 |

Note: Model 1: no adjustment for confounding factors; Model 2: adjusted for confounding factors such as age, hypertension, diabetes, Fazekas score, TC, LDL, and HCY; Model 3: Lp-PLA2 as a continuous variable, adjusted for confounding factors such as age, hypertension, diabetes, Fazekas score, TC, LDL, and HCY.

According to the ROC curve (Figure 1), the optimal cut-off value of Lp-PLA2 that predicted CMBs was 184.36 ng/ml, which yielded a sensitivity of 69.0%, a specificity of 60.2%, and an AUC of 0.664 (95% CI: 0.587-0.741, *P* = 0.000).


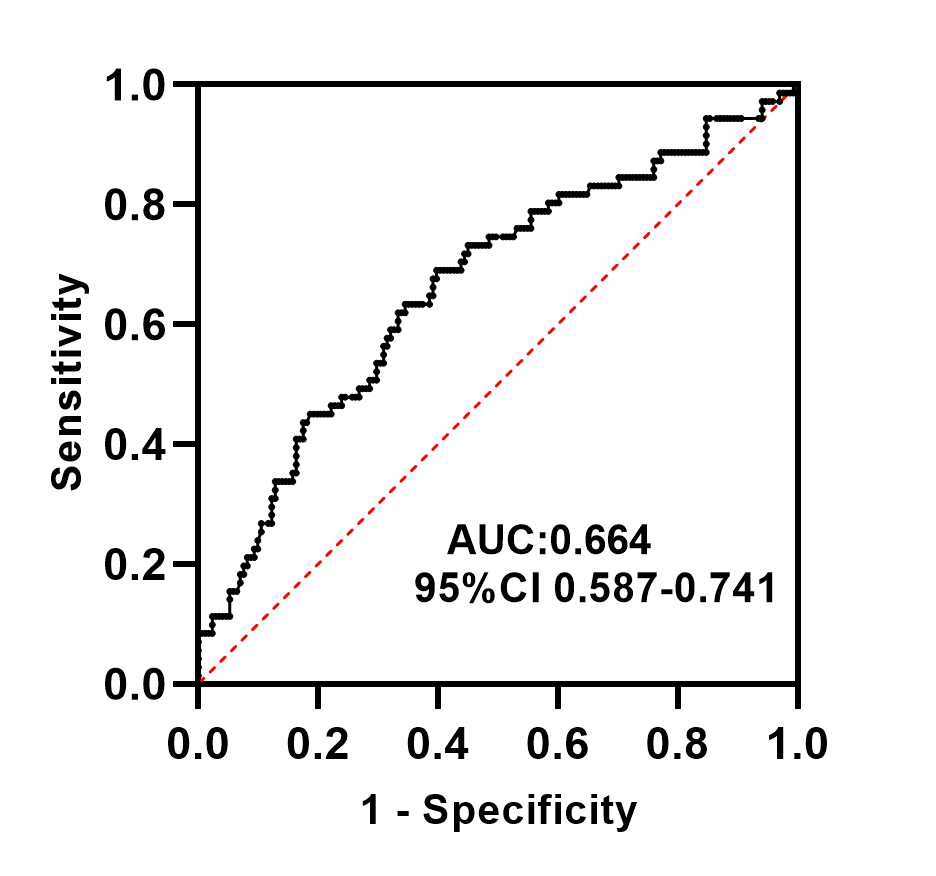


**Fig 1** ROC curve for Lp-PLA2 levels and CMBs

## Discussion

The purpose of this study was to investigate the correlation between Lp-PLA2 levels and CMBs in patients with AIS. We found that a high level of Lp-PLA2 was a potential risk factor for CMBs and determined that the optimal cut-off value of Lp-PLA2 that predicted CMBs was 184.36 ng/ml according to the ROC curve.

Of the included patients with AIS, 29.3% (71/242) had CMBs; this proportion is slightly lower than the 34% observed in previous studies[2]. This difference may be due to differences in the MRI sequences used among studies. T2-weighted gradient echo sequence (T2*GRE) and SWI are the most commonly used methods to identify CMBs[14]. Compared with T2*GRE, SWI has a much higher spatial resolution and is more sensitive to iron atoms in haemosiderin[15, 16]; it is thus the most reliable method of detecting CMBs to date. In our study, SWI was used to detect CMBs, which excluded other imaging findings and decreased the false-positive rate.

Lp-PLA2 has emerged as an inflammatory marker; it is mainly secreted by inflammatory cells such as macrophages and monocytes[17]. The expression of Lp-PLA2 is regulated by proinflammatory factors, such as IL-6, TNF-α, colony stimulating factor, and glucocorticoids[18]. Studies have demonstrated that Lp-PLA2 plays a role in the initiation, formation, development and rupture of atherosclerosis[19, 20]. Lp-PLA2 is also an independent risk factor for ischaemic stroke[21], predicting the occurrence, development and recurrence risk[22-24], and an independent marker of poor prognosis of stroke patients. The mechanism underlying this relationship is as follows: Lp-PLA2 combines with LDL through apolipoprotein B100 and promotes the condensation and oxidation of LDL to form oxidized LDL (oxLDL), and by hydrolysing the phospholipid components in oxLDL, lipid proinflammatory substances such as lysophosphatidylcholine (LPC) and oxidized free fatty acids (oxFFAs) are produced[25]. Proinflammatory substances further damage vascular endothelial cells, causing vascular dysfunction and leading to atherosclerosis and stroke. Studies have suggested that the blood‒brain barrier(BBB) is a downstream product of endothelial cells[26]. Therefore, it can be speculated that Lp-PLA2 and its products damage the BBB, leading to an increase in its permeability allowing harmful toxins and immune cells to enter the brain and changing in the structure of small blood vessels[27], leading to the formation of CMBs.

To date, a few studies have explored the correlation between Lp-PLA2 levels and CMBs, but they have not reached unified conclusion. A study of 819 community-living seniors[28] found that Lp-PLA2 levels were not significantly correlated with CMBs, but in patients with apolipoprotein (APOE) ε2 or ε4 alleles, deep CMBs were closely associated with high levels of Lp-PLA2. In contrast, a prospective study in China[29] showed that the Lp-PLA2 levels in the CMB group were significant higher than those in the normal control group (*P* < 0.05), indicating that there was a correlation between Lp-PLA2 levels and CMBs. Our study indicated that the Lp-PLA2 levels in the CMB group were significantly higher than those in the no-CMB group. Using the 1^st^ quartile of Lp-PLA2 levels (the lowest levels) as the reference group, univariate logistic regression analysis showed that the 4^th^ quartile of Lp-PLA2 levels (the highest levels) had a higher risk of CMBs (OR = 1.460, 95% CI: 1.188-1.795, *P* = 0.000), and this association persisted after adjusting for relevant risk factors (OR = 1.370, 95% CI: 1.096-1.713, *P* = 0.006). In addition, when Lp-PLA2 was used as a continuous variable for logistic regression analysis, the level of Lp-PLA2 was also correlated with CMBs after adjusting for relevant risk factors (OR = 1.014, 95% CI: 1.005-1.024, *P* = 0.004). Thus, a high level of Lp-PLA2 was a potential risk factor for CMBs in patients with AIS. This conclusion is not completely consistent with the results of the above two studies, possibly due to differences in the enrolled population, CMB detection equipment and Lp-PLA2 measurement methods.

Furthermore, the 2012 guidelines of the American Society of Endocrinologists recommend using a cut-off value of Lp-PLA2 concentration ≥ 200 ng/ml, which is associated with a higher risk of cardiovascular events (< 200 ng/ml is considered the normal range)[30]. However, some researchers have suggested that the threshold level of Lp-PLA2 varies according to sex and race[31]. Subsequently, a Chinese study reported that the cut-off value of Lp-PLA2 that predicted a risk of cardiovascular events was 159 ng/mL[32]. However, after reviewing the relevant literature, there are no reports on the cut-off value of Lp-PLA2 that predicts the risk of CMBs. In this study, the ROC curve was used to determine the optimal cut-off value of Lp-PLA2 that predicted CMBs; an Lp-PLA2 level > 184.36 ng/ml indicates a higher risk of CMBs.

Nevertheless, this study has several limitations. First, our study was performed at a single centre, which could lead to selection bias and reduce the study's generalizability. Second, we only evaluated the plasma Lp-PLA2 level at admission and did not conduct dynamics observation of the Lp-PLA2 level; thus, our methods may not have been sufficiently comprehensive. Finally, this study was an observational study, which can only describe a correlation between Lp-PLA2 levels and CMBs but cannot confirm a causal relationship. Therefore, interpretation of our data merits caution, and larger prospective cohort studies are required to confirm the causal relationship between Lp-PLA2 levels and CMBs in the future.

In summary, we found that a high level of Lp-PLA2 was a potential risk factor for CMBs in patients with AIS and established a (preliminary) optimal cut-off value of Lp-PLA2 that predicted CMBs at 184.36 ng/ml. Therefore, patients with AIS whose Lp-PLA2 level is greater than 184.36 ng/ml warrant timely screening for CMBs, delay or reduce the occurrence of CMBs by controlling risk factors, so as to reduce the risk of stroke occurrence and recurrence and improve the prognosis of stroke.

**Abbreviations**

CMBs: Cerebral microbleeds; Lp-PLA2: Lipoprotein-associated phospholipase A2; AIS: Acute Ischaemic Stroke; SWI: Susceptibility Weighted Imaging; WMLs: White matter lesions; ROC: Receiver operating characteristic curve; Hs-CRP: high-sensitivity C-reactive protein; FPG: fasting plasma glucose; TG: triglyceride; TC: total cholesterol; LDL-C: low-density lipoprotein cholesterol; HDL: high-density lipoprotein; Cr: creatinine; PT: prothrombin time; APTT: activated partial thromboplastin time; INR: International Normalized Ratio; FIB: fibrinogen; Hcy:homocysteine; AUC: the area under the curve.

**Acknowledgements** We would like to thank all the researchers and patients who participated in this study, especially two neuroradiologists, Wanhong Chen and Yong Liu.

**Author Contributions** Xiaojiu Zhang: designed the study, conducted the statistical analysis, and drafted the article.Lu Liu and Nan Jiang: contributed with data acquisition and analysis.Yufeng Liu and Qing Wang : Collected patient clinical baseline data and imaging data. Xiaohong Tang: revised the manuscript. Qijin Zhai: revised the manuscript and supervised this study.Liandong Zhao:participated in conception and design of the study, supervised this study,and revised the manuscript.All authors read and approved the final manuscript.

**Funding**

Not applicable.

**Availability of data and materials**

The datasets generated and analysed during the current study are not publicly available due to data protection of the patients and their relatives but are available from the corresponding author on reasonable request.

## Declarations

**Ethics approval and consent to participate**

The study protocol was approved by the Ethical Committee of Huai'an Hospital affiliated with Xuzhou Medical University. This study was performed in line with the principles of the Declaration of Helsinki.

**Consent for publication**

Not applicable.

**Competing interest**

The authors declare that they have no competing interests.

**References**

1. Viswanathan A, Chabriat H. Cerebral microhemorrhage. Stroke. 2006;37(2):550-5.

2. Cordonnier C, Al-Shahi Salman R, Wardlaw J. Spontaneous brain microbleeds: systematic review, subgroup analyses and standards for study design and reporting. Brain. 2007;130(Pt 8):1988-2003.

3. Charidimou A, Shams S, Romero JR, et al. Clinical significance of cerebral microbleeds on MRI: A comprehensive meta-analysis of risk of intracerebral hemorrhage, ischemic stroke, mortality, and dementia in cohort studies (v1). Int J Stroke. 2018;13(5):454-68.

4. Wilson D, Ambler G, Lee KJ, et al. Cerebral microbleeds and stroke risk after ischaemic stroke or transient ischaemic attack: a pooled analysis of individual patient data from cohort studies. Lancet Neurol. 2019;18(7):653-65.

5. Xu CX, Xu H, Yi T, Yi XY, Ma JP. Cerebral Microbleed Burden in Ischemic Stroke Patients on Aspirin: Prospective Cohort of Intracranial Hemorrhage. Front Neurol. 2021;12:742899.

6. Capuana ML, Lorenzano S, Caselli MC, Paciaroni M, Toni D. Hemorrhagic risk after intravenous thrombolysis for ischemic stroke in patients with cerebral microbleeds and white matter disease. Neurol Sci. 2021;42(5):1969-76.

7. Gu Y, Gutierrez J, Meier IB, Guzman VA, Manly JJ, Schupf N, Brickman AM, Mayeux R. Circulating inflammatory biomarkers are related to cerebrovascular disease in older adults. Neurol Neuroimmunol Neuroinflamm. 2019;6(1):e521.

8. Low A, Mak E, Rowe JB, Markus HS, O&#39, Brien JT. Inflammation and cerebral small vessel disease: A systematic review. Ageing Res Rev. 2019;53:100916.

9. Liang Q, Lei X, Huang X, Fan L, Yu H. Elevated Lipoprotein-Associated Phospholipase A2 is Valuable in Prediction of Coronary Slow Flow in Non-ST-Segment Elevation Myocardial Infarction Patients. Curr Probl Cardiol. 2021;46(3):100596.

10. Hatano S. Experience from a multicentre stroke register: a preliminary report. Bull World Health Organ. 1976;54(5):541-53.

11. Wardlaw JM, Smith EE, Biessels GJ, et al. Neuroimaging standards for research into small vessel disease and its contribution to ageing and neurodegeneration. Lancet Neurol. 2013;12(8):822-38.

12. Adachi M, Sato T. Characterization of the Growth of Deep and Subcortical White Matter Hyperintensity on MR Imaging: A Retrospective Cohort Study. Magn Reson Med Sci. 2017;16(3):238-44.

13. Fazekas F, Chawluk JB, Alavi A, Hurtig HI, Zimmerman RA. MR signal abnormalities at 1.5 T in Alzheimer's dementia and normal aging. AJR Am J Roentgenol. 1987;149(2):351-6.

14. Wach-Klink A, Iżycka-Świeszewska E, Kozera G, Sobolewski P. Cerebral microbleeds in neurological practice: concepts, diagnostics and clinical aspects. Neurol Neurochir Pol. 2021;55(5):450-61.

15. Shao L, Wang M, Ge XH, Huang HD, Gao L, Qin JC. The use of susceptibility-weighted imaging to detect cerebral microbleeds after lacunar infarction. Eur Rev Med Pharmacol Sci. 2017;21(13):3105-12.

16. Hageman G, Hof J, Nihom J. Susceptibility-Weighted MRI and Microbleeds in Mild Traumatic Brain Injury: Prediction of Posttraumatic Complaints. Eur Neurol. 2022:1-9.

17. Lerman A, McConnell JP. Lipoprotein-associated phospholipase A2: a risk marker or a risk factor. Am J Cardiol. 2008;101(12A):11F-22F.

18. Silva IT, Mello AP, Damasceno NR. Antioxidant and inflammatory aspects of lipoprotein-associated phospholipase A₂ (Lp-PLA₂): a review. Lipids Health Dis. 2011;10:170.

19. Zheng H, Cui D, Quan X, Yang W, Li Y, Zhang L, Liu E. Lp-PLA2 silencing protects against ox-LDL-induced oxidative stress and cell apoptosis via Akt/mTOR signaling pathway in human THP1 macrophages. Biochem Biophys Res Commun. 2016;477(4):1017-23.

20. Fatemi S, Gottsäter A, Zarrouk M, Engström G, Melander O, Persson M, Acosta S. Lp-PLA(2) activity and mass and CRP are associated with incident symptomatic peripheral arterial disease. Sci Rep. 2019;9(1):5609.

21. Hu G, Liu D, Tong H, Huang W, Hu Y, Huang Y. Lipoprotein-Associated Phospholipase A2 Activity and Mass as Independent Risk Factor of Stroke: A Meta-Analysis. Biomed Res Int. 2019;2019:8642784.

22. Wei L, Ke Z, Zhao Y, Cai Z. The elevated lipoprotein-associated phospholipase A2 activity is associated with the occurrence and recurrence of acute cerebral infarction. Neuroreport. 2017;28(6):325-30.

23. Wang Y, Hu S, Ren L, Lei Z, Lan T, Cai J, Li C. Lp-PLA(2) as a risk factor of early neurological deterioration in acute ischemic stroke with TOAST type of large arterial atherosclerosis. Neurol Res. 2019;41(1):1-8.

24. Li X, Xu L, Xu Z. The diagnostic and prognostic performance of Lp-PLA2 in acute ischemic stroke. Med Clin (Barc). 2021;156(9):437-43.

25. Bonnefont-Rousselot D. [Lp-PLA2, a biomarker of vascular inflammation and vulnerability of atherosclerosis plaques]. Ann Pharm Fr. 2016;74(3):190-7.

26. Saint-Pol J, Gosselet F, Duban-Deweer S, Pottiez G, Karamanos Y. Targeting and Crossing the Blood-Brain Barrier with Extracellular Vesicles. Cells. 2020;9(4).

27. Wadi LC, Grigoryan MM, Kim RC, Fang C, Kim J, Corrada MM, Paganini-Hill A, Fisher MJ. Mechanisms of Cerebral Microbleeds. J Neuropathol Exp Neurol. 2020;42(2):1093-9.

28. Romero JR, Preis SR, Beiser AS, et al. Lipoprotein phospholipase A2 and cerebral microbleeds in the Framingham Heart Study. Stroke. 2012;43(11):3091-4.

29. Liu Y, Liu X, Zhang X, Hou C, Tang P. Study on the Relationship between Lipoprotein-related Phospholipase A2,Homocysteine,Beta-N-1-Deoxy Fructosyl Component of Hemoglobin and Cerebral Micobleeds. Journal of Modern Laboratory Medicine. 2017;32(6):35-8.

30. Jellinger PS, Smith DA, Mehta AE, Ganda O, Handelsman Y, Rodbard HW, Shepherd MD, Seibel JA. American Association of Clinical Endocrinologists' Guidelines for Management of Dyslipidemia and Prevention of Atherosclerosis. Endocr Pract. 2012;18 Suppl 1:1-78.

31. Ntzouvani A, Giannopoulou E, Fragopoulou E, Nomikos T, Antonopoulou S. Energy Intake and Plasma Adiponectin as Potential Determinants of Lipoprotein-Associated Phospholipase A(2) Activity: A Cross-Sectional Study. Lipids. 2019;54(10):629-40.

32. Xi H, Cheng GL, Hu FF, Li SN, Deng X, Zhou Y. The Use of Lipoprotein-Associated Phospholipase A2 in a Chinese Population to Predict Cardiovascular Events. Biomed Environ Sci. 2022;35(3):206-14.
